# Tools for Developing, Implementing, and Evaluating State Policy

**Published:** 2008-03-15

**Authors:** Bernadette Ford Lattimore, So O'Neil, Melanie Besculides

**Affiliations:** Division for Heart Disease and Stroke Prevention, National Center for Chronic Disease Prevention and Health Promotion, Centers for Disease Control and Prevention; Mathematica Policy Research, Inc, Princeton, New Jersey; Mathematica Policy Research, Inc, Princeton, New Jersey

## Abstract

Policy can improve health by initiating changes in physical, economic, and social environments. In contrast to interventions focused on individual people, policies have the potential to affect health across populations. For this reason, the Division for Heart Disease and Stroke Prevention of the Centers for Disease Control and Prevention (CDC) advises states funded under the Heart Disease and Stroke Prevention Program to engage in activities supporting the development and maintenance of policies that can help reduce the burden of cardiovascular disease.

Currently, the Division for Heart Disease and Stroke Prevention funds programs in 33 states and the District of Columbia to promote cardiovascular health. One goal of these programs is to build states' capacity to develop, implement, track, and sustain population-based interventions that address heart disease and stroke. Because of the critical role of policy in these activities, CDC provides guidance in developing, implementing, and evaluating policy. In 2004, the division contracted with Mathematica Policy Research, Inc, to conduct the Heart Disease and Stroke Prevention Policy Project, which included development of an online database of state heart disease and stroke prevention policies and a mapping application to show which states have these policies.

We discuss the method for developing the database, mapping application, and other tools to assist states in developing, implementing, and evaluating heart disease and stroke prevention policies. We also highlight lessons learned in developing these tools and ways that states can use the tools in their policy and program planning.

## Introduction

Policies, which consist of laws, regulations, and rules, can determine how organizations providing health services are funded, organized, or held accountable and can change physical, economic, and social environments (1). As a result, a policy is a type of intervention that can significantly affect health over the long term. Legislation establishing smoke-free policies is a good example. Secondhand smoke, a known carcinogen, causes 35,000 deaths from heart disease and 3,000 deaths from lung cancer annually among nonsmokers in the United States (2). Knowledge of these statistics led to smoke-free policies in a number of states, with smoking banned within entire venues. Exposure to secondhand smoke soon decreased sharply, in part because of social and environmental changes brought about by these policies.

To provide leadership in improving cardiovascular health nationwide, reducing cardiovascular disease, and eliminating disparities in heart disease and stroke, Congress mandated in 1998 the creation of the Cardiovascular Health Branch of the Centers for Disease Control and Prevention (CDC). To help support its mission, the branch initiated a national, state-based heart disease and stroke prevention (HDSP) program with funding for eight states. The branch became the Division for Heart Disease and Stroke Prevention in January 2006. Currently, state HDSP programs exist in 33 states and the District of Columbia. These state programs address six priority areas established by the division: controlling high blood pressure, controlling high blood cholesterol, increasing awareness of the signs and symptoms of heart attack and stroke, improving emergency response, improving quality of care, and eliminating health disparities. To measure progress in these areas, the programs monitor cardiovascular disease and its related risk factors and assess policy and environmental support for the prevention of heart disease and stroke in their individual states. A new funding cycle that began June 30, 2007, supports the same number of states (but not all of the same states) and the District of Columbia.

As outlined in its funding requirements, the Division for Heart Disease and Stroke Prevention emphasizes the need for policies aimed at preventing heart disease and stroke. Feedback from state programs, however, revealed a need for more research documenting the effectiveness of different prevention policies to help determine how best to channel their limited resources. This type of research can be difficult for states to conduct on a large scale themselves because they often lack the resources or technical expertise to evaluate policy interventions.

To answer this need, the division contracted with Mathematica Policy Research, Inc (MPR), in 2004 to conduct the HDSP Policy Project for the purpose of developing an annotated bibliography of state HDSP policy sources; a centralized, online database of policies from all 50 states and the District of Columbia; a mapping application indicating where HDSP policies exist; a guide outlining the fundamentals of HDSP policy making; and a handbook on using an adapted RE-AIM (www.re-aim.org) framework to assess these policies (3). RE-AIM, which stands for the five components of an evaluation framework (reach, efficacy, adoption, implementation, and maintenance), is commonly used to systematically evaluate interventions for changing health behaviors. These products can help states understand the policy process and easily access state HDSP policy information. With the database, a state can decide which policies are applicable and replicable in its specific jurisdictions and track policies over time. State HDSP programs can also find guidance on developing, implementing, and evaluating policies to prevent cardiovascular disease.

In developing the HDSP Policy Project, CDC and MPR conducted a comprehensive literature review, a focus group with representatives of state HDSP programs, and interviews with policy experts. To guide the process, we convened an advisory panel of experts in the field of heart disease and stroke and in policy and environmental interventions. Members were from government, health care, and advocacy organizations. This article describes the creation of the online tools and related products and highlights the challenges and lessons learned in conducting a project of this magnitude.

## The Online HDSP Policy Database and Mapping Application

### Literature review and annotated bibliography

The project began with an extensive literature review that took place from September 2004 through June 2005 and resulted in an annotated bibliography database, created in ProCite (Thomson Corporation, Stamford, Connecticut), that contained 174 sources of HDSP state policies and activities. The sources included reports, Web sites, newsletters, guidelines, and press releases related to the prevention of heart disease and stroke. We excluded policies dealing specifically with cardiovascular disease risk factors because policy databases already exist for these. A link on the HDSP Policy Project Web site takes users to a bibliography of the original sources of the policies in the database.

### Web site

From sources gathered in the annotated bibliography database, we identified 207 policies to populate the HDSP policy database. The policies we found were in force either from 1978 through 2005 or at some time during that period. To house the policies systematically, we created an Excel (Microsoft Corporation, Redmond, Washington) spreadsheet based on the following information:

- State
- Bill number
- Status (whether a bill is enacted or is current law [An enacted bill has been passed through the legislature and signed by the governor but has not necessarily become part of the state legal code; a current law has become part of the legal code.])
- Year (when the bill was passed)
- Topic area
- CDC priority area
- Policy abstract
- PDF file name of policy

After completing the database, we created a Web site using as a template the code from the legislative database Web site of CDC's Division of Nutrition, Physical Activity, and Obesity. Microsoft SQL Server (Microsoft Corporation, Redmond, Washington) was used to design and maintain the Web site. The HDSP policy database and user's guide, a list of frequently asked questions, CDC contact information, a site map, and links to additional resources are included to assist users. An administrative Web site, available only to authorized personnel, allows for updating or modifying the database when new resources become available.

### Mapping application

**Figure 1.**
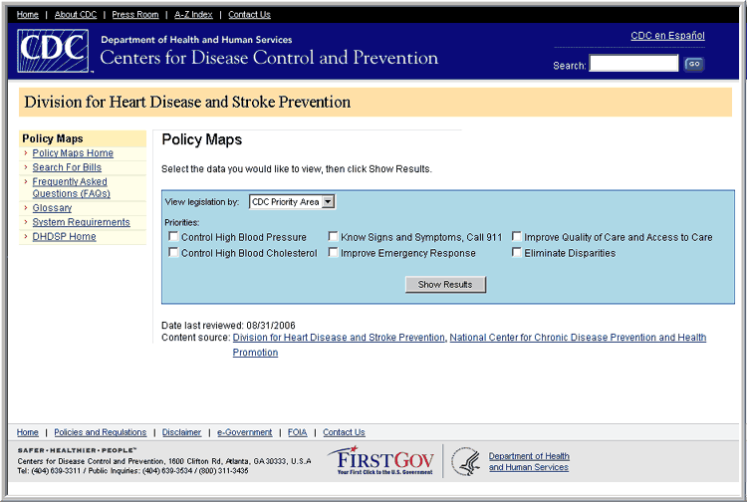
Home page of the mapping application for the Heart Disease and Stroke Prevention Policy Project, Centers for Disease Control and Prevention, United States.

**Figure 2.**
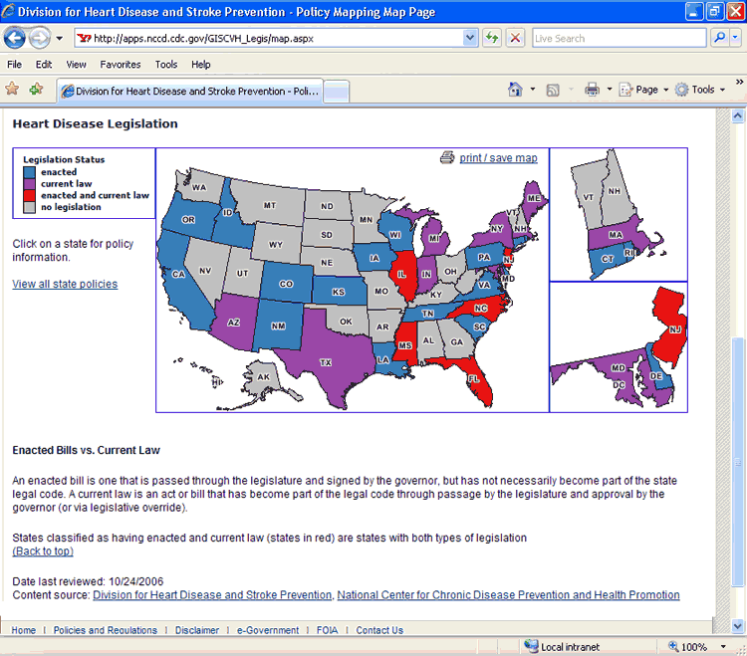
Web page indicating states with heart disease legislation for 1978–2005, Heart Disease and Stroke Prevention Policy Project, Centers for Disease Control and Prevention, United States.

**Figure 3.**
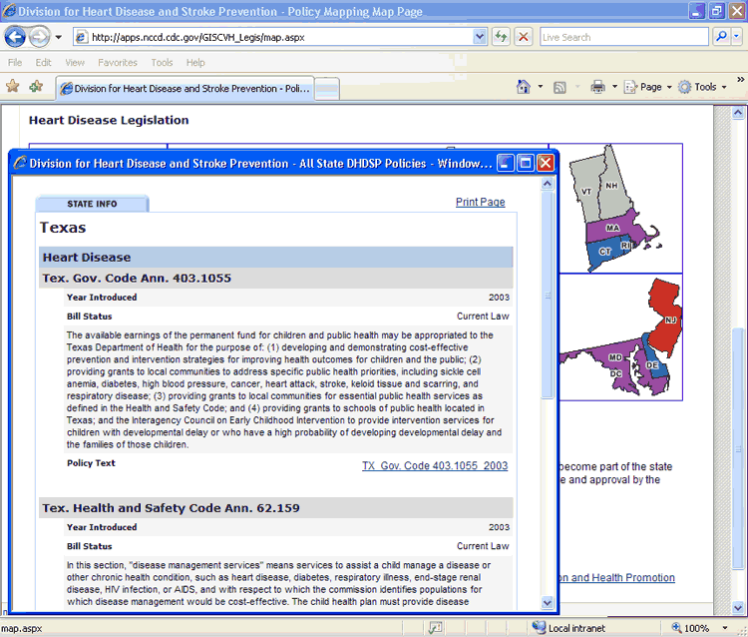
Web page detailing heart disease legislation for Texas, Heart Disease and Stroke Prevention Policy Project, Centers for Disease Control and Prevention, United States.

The mapping application allows users to see the distribution of enacted or current HDSP policies across the United States (Figure 1). To begin the process, CDC teamed with experts in geographic information systems from Northrop Grumman Corporation, including a usability engineer who pilot tested the application. Users can view legislation by CDC priority area or by topic. For example, if the user selects heart disease as the search topic, a color-coded map of the United States appears (Figure 2) showing which states have enacted laws, current laws, both enacted and current laws, or no legislation related to heart disease. The user can then click on a state and view information about the legislation, including the year it was introduced, its status, a summary of the policy, and a link to the legislation in its entirety (Figure 3).

## Other Policy Tools

### Guide to policy making

CDC and MPR used information from the focus groups and interviews with policy experts to create the *Guide to the ABCs of State Heart Disease and Stroke Policymaking* (4). Highlights of the guide include an outline of the policy-making process, the role of state and local health departments in developing policy, challenges to implementing policy, and the reasons that policy evaluation is not commonly written into legislation. The following key findings emerged:

- Mandates to enact and enforce regulations should be adequately funded.
- Policy should be developed at the community level to gain local support that can then spread to the rest of the state.
- Partnerships with stakeholders should be created to help advocate for HDSP policies.
- Limitations in data can hinder policy evaluation.

The guide can be used in numerous ways. For example, a new HDSP program manager who is unfamiliar with how policies are developed in the state can go to the guide for the tools needed to effectively participate in policy development. For a task force on stroke, the guide offers information about the challenges of implementing policy in that area.

### Handbook on policy assessment

Because illustrating outcomes of policies can be helpful to state programs and other stakeholders, CDC and MPR created *Assessing Heart Disease and Stroke Prevention Policies With the RE-AIM Framework*. The handbook outlines the assessment of selected policies from each of the six CDC priority areas using an adapted version of the RE-AIM framework (3). We used RE-AIM because its system-based and social-ecologic components allow for the assessment of interventions at multiple levels. Linking long-term outcomes to a policy proved difficult, however, because most HDSP policies are too recent to have measurably influenced long-term outcomes such as changes in health status. We, therefore, adapted the RE-AIM framework to capture short-term outcomes, such as policy implementation, and intermediate-term outcomes, such as increased program surveillance (Table).

To collect the data needed to assess the seven selected policies, we reviewed, recorded, and filed information needed to use the RE-AIM framework; developed interview guides for each of the seven policies; and interviewed, primarily by telephone, the experts whom we had identified. We organized interview notes within the parameters of the RE-AIM framework to facilitate analysis. The resulting handbook contains an analysis and summary table for each policy and the methods used to apply RE-AIM to the assessments and discusses challenges in using RE-AIM to assess policies. The following key findings emerged:

- Reporting and collecting data are essential to improving ongoing activities and assessing the outcomes of a policy.
- Legislative support is necessary to ensure the passage of policy, funding for implementing and maintaining policies, and the success of activities resulting from a policy.
- A strong evidence base increases the chances that a policy will be adopted and implemented.
- Involvement of different stakeholders, including consumers, providers, and legislators, in policy making ensures that policies are smoothly adopted, implemented, and maintained.

The handbook provides a method of assessing policies in the event that resources are unavailable to evaluate a mandated policy. For example, suppose a policy to create a stroke task force was passed 5 years ago without any funding for evaluation. The state HDSP program could consult the handbook to find out how to adapt the RE-AIM framework to assess the policy and could then use the assessment to determine what changes would potentially increase the effectiveness of the task force.

## Lessons Learned

The following lessons were learned from the HDSP Policy Project:

- An advisory panel can provide valuable insight, ideas, and resources for a project. Contributions of this project's advisory panel included reviewing drafts of the documents and providing key considerations for assessing policies.
- Having a mapping application in addition to an online database is helpful for users who are visual learners or who find printed maps valuable when communicating with stakeholders.
- The RE-AIM framework may be adapted to assess policies. Although the process is challenging, focusing measurement on the outcomes stated in legislation provides clear parameters for the adaptation. Validation of the adaptation is an area for future research.
- Creating logic models to provide an organized visual representation of short-term, intermediate, and long-term outcomes is crucial and allows for systematic assessment.
- Policy evaluation is time-sensitive. The less time between a policy's implementation and its evaluation, the less information is available about the effect of the policy.

## Conclusion

The 2-year HDSP Policy Project provided stakeholders with important tools for supporting HDSP policy activities in their states. These products are available at www.cdc.gov/dhdsp/dhdspleg and from CDC's Division for Heart Disease and Stroke Prevention Web site (www.cdc.gov/dhdsp). CDC plans to update the database annually. With these tools, funded state programs and other stakeholders can gain insight into policy, its application, and its impact on heart disease and stroke.

## Figures and Tables

**Table:** Application of the RE-AIM Framework for Assessing Interventions to the Assessment of Policies in the Heart Disease and Stroke Prevention (HDSP) Policy Project, Centers for Disease Control and Prevention, United States, 2006

RE-AIM Model Component	Definition	Application to HDSP Policy Project
Reach	An individual measure of the percentage and risk characteristics of people who are affected by a policy or program.	Evidence that a policy targets specific populations and settings and that a plan or intervention is in place to reach the targeted populations.
Efficacy	An individual measure of positive and negative consequences of a program for four types of outcomes: behavioral, quality-of-life, physiological, and satisfaction of participants.	Evidence of a method to track cardiovascular disease and risk factors and the development of indicators of policy and environmental change.
Adoption	An organization- and community-level measure of the proportion and representativeness of settings (e.g., worksite, health departments, or communities) that adopt a given policy or program.	Evidence that a policy was instituted in the intended settings (e.g., community, health care, worksite, school) or in a wide variety of settings.
Implementation	An organization- and community-level measure of the extent to which an intervention is implemented as intended.	Evidence of intended interventions or activities resulting from the policy.
Maintenance	An individual-, organization-, and community-level measure of the extent to which an intervention is sustained over time and becomes a relatively stable, enduring part of behavior.	Evidence of efforts to sustain and evaluate interventions or activities resulting from the policy.
